# Alpha Helices Are More Robust to Mutations than Beta Strands

**DOI:** 10.1371/journal.pcbi.1005242

**Published:** 2016-12-09

**Authors:** György Abrusán, Joseph A. Marsh

**Affiliations:** 1 MRC Human Genetics Unit, Institute of Genetics and Molecular Medicine, University of Edinburgh, Western General Hospital, Crewe Road, Edinburgh EH4 2XU, United Kingdom; 2 Institute of Biochemistry, Biological Research Centre of the Hungarian Academy of Sciences, Szeged, Temesvári krt. 62, Hungary; University College London, UNITED KINGDOM

## Abstract

The rapidly increasing amount of data on human genetic variation has resulted in a growing demand to identify pathogenic mutations computationally, as their experimental validation is currently beyond reach. Here we show that alpha helices and beta strands differ significantly in their ability to tolerate mutations: helices can accumulate more mutations than strands without change, due to the higher numbers of inter-residue contacts in helices. This results in two patterns: a) the same number of mutations causes less structural change in helices than in strands; b) helices diverge more rapidly in sequence than strands within the same domains. Additionally, both helices and strands are significantly more robust than coils. Based on this observation we show that human missense mutations that change secondary structure are more likely to be pathogenic than those that do not. Moreover, inclusion of predicted secondary structure changes shows significant utility for improving upon state-of-the-art pathogenicity predictions.

## Author Summary

The factors that determine the robustness and evolvability of proteins are still largely unknown. In this work the authors show that different secondary structure elements of proteins (helices and strands) differ in their ability to tolerate mutations, and demonstrate that it is caused by differences in the number of non-covalent residue interactions within these secondary structure units. The results suggest that engineering de novo all-alpha proteins should be easier than all-beta ones, as more sequences can to fold to the same topology. Additionally, secondary structure can be used to improve current methods of pathogenicity predictions; mutations that change secondary structure are more likely to be pathogenic than mutations that do not, due to their strong destabilizing effect on protein structure.

## Introduction

In recent years, genome sequencing studies have uncovered an enormous amount of human genetic variation, both in coding and noncoding regions of the human genome. As a consequence, developing computational methods that accurately predict whether mutations have any phenotypic or pathogenic consequences is a major goal of bioinformatics, and a number of tools have been developed to address this problem [1], however they currently achieve only a limited accuracy [2,3].

After evolutionary conservation, protein structural information is one of the most useful predictors of the phenotypic effects of missense mutations. Missense mutations may disrupt protein structure and function at least in two ways: either by destabilizing the entire protein fold [4,5], or by modifying functional residues, i.e. active sites or protein-protein interactions [6], and pathogenic mutations are enriched in both the buried cores of proteins [7] and in protein interfaces [8]. The factors that make protein folds stable, i.e. robust against mutations, have been studied in an evolutionary context, as robustness against mutations, and evolutionary innovability are related concepts: protein folds that tolerate mutations better are more likely to evolve functional innovations [9]. It has been suggested that the key structural property of proteins that determines their ability to accept a mutation without destabilizing the fold is the density of contacts between residues [10,11] (measured either with the length-normalized number of contacts [11], or the largest eigenvalue of contact density matrix [10]), and the higher the contact density of a given fold, the more robust it is against mutations. Subsequent studies have demonstrated the validity of the concept both experimentally and also through comparative analyses, showing that more stable proteins are more likely to accept destabilizing mutations [12,13], and that the number of sequences that fold into a particular SCOP (Structural Classification Of Proteins) domain, and their evolutionary rate, is positively correlated with the contact density of the fold [14,15].

Previous work on mutational robustness, i.e. the ability to accept mutations without change, has focused mostly on protein tertiary structure. Here we have considered secondary structure, investigating whether protein regions with different secondary structure differ in their robustness against mutations, as suggested by a previous, preliminary study by one of us [16]. We performed a large-scale analysis of SCOP [17] domains and the Protein Data Bank (PDB), and show that alpha helices are more robust than beta strands, i.e. can tolerate more sequence change without changing secondary structure. This appears to be primarily due to the higher number of residue interactions in helices, and both helices and strands are more robust than regions with no secondary structure (coils). Using currently available data of human variation and disease, we also tested whether this is reflected in the distribution of pathogenic missense mutations, and found that mutations which change secondary structure are much more likely to be pathogenic than mutations that do not. Finally, we find that information on whether a mutation is likely to disrupt secondary structure can be used to improve predictions of pathogenicity.

## Results and Discussion

### Helices can accumulate more mutations than strands or coils

**Fig 1:**
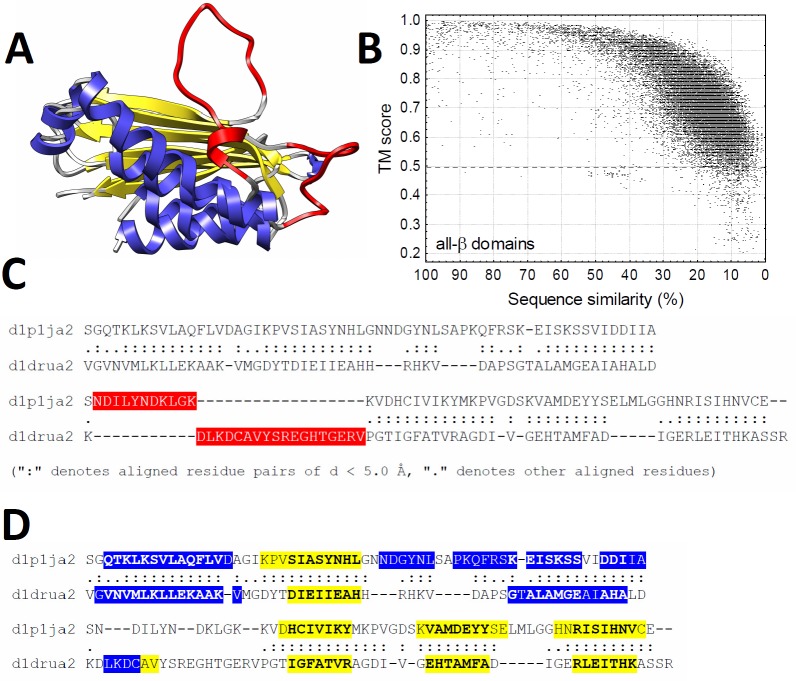
The outline of the comparative analyses. **A)** For all domains of every SCOP class, pairwise structural alignments were created with TMalign (blue–helices, yellow–strands, red–unaligned regions). **B)** Structural alignments with TM score below 0.5 were excluded from the analysis, and the pairwise alignments were ordered according to the sequence similarity of the aligned structures. **C)** Structurally unaligned regions (red) were refined with Rascal, resulting in high quality pairwise alignments. **D)** In the pairwise alignments the secondary structure, RSA and contact density were determined for each residue.

We tested whether helices are more robust to mutations than strands using the four main classes of SCOP domains: all-α, all-β, α/β and α+β domains (all-α domains contain only helices, all-β domains contain only strands, α+β domains contain both helices and strands that are segregated within the domains, while α/β domains contain alternating helices and strands). We used a comparative method (Fig 1); first, we made all possible pairwise structural alignments between all domains within all SCOP families with TMalign [18]; next, we determined the secondary structure of the domains in the alignments, and examined how secondary structure similarity (the percent of aligned helix residues that remained helices in both proteins) changes with sequence similarity (Fig 1). Additionally, we determined the relative solvent accessible area (RSA) for each residue of the alignments, and the frequency of indels in the alignments (S1 Fig and S2 Fig).

**Fig 2:**
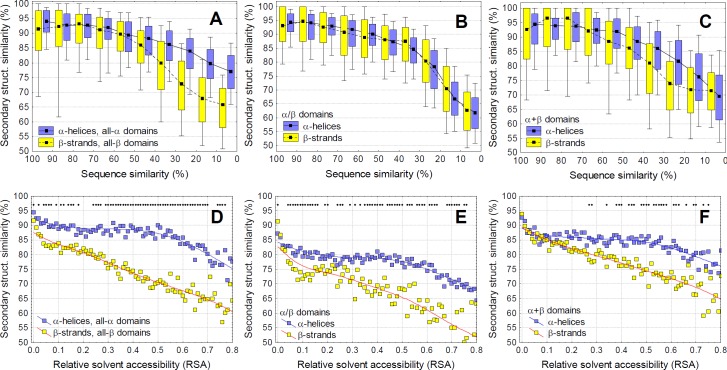
Alpha helices can accept more mutations than beta strands. **A-C)** Secondary structure similarity of pairwise alignments as a function of sequence similarity. Pairwise alignments were grouped into 10% bins based on their sequence similarity. α-helices change significantly less with sequence change than beta strands in case of all-α, all-β and α+β domains. **D-F)** Since in α/β and α+β domains there is a large difference in the buriedness of helices and strands (see S1 Fig), using alignments with 10–20% similarity, we added relative solvent accessibility (RSA) as a covariate. When this correction is applied (i.e. the different levels of buriedness are taken into account), residues of helices are significantly more robust for mutations than strands in all SCOP classes, except for the most buried residues with RSA < 0.1 (diamonds indicate significant difference, tests of proportions, p < 0.05 after Holm-Bonferroni correction).

Overall, our results indicate that alpha helices can accumulate significantly more mutations than beta strands without change in the structure (Fig 2), and both helices and strands change slower than coils (S3 Fig). With decreasing sequence similarity, secondary structure similarity decreases significantly faster in all-β than all-α domains (Fig 2A, p < 2.2 x 10^−16^, ANCOVA, using alignments with 10–30% sequence similarity), and also within α+β domains (Fig 2C, p < 2.2 x 10^−16^, ANCOVA), even without taking into account the very different relative solvent accessibility (RSA) of helices and strands in these proteins (see S1 Fig).

As residues close to the surface accumulate mutations and change faster than the core [19,20], we also calculated secondary structure similarity for residues with different RSA, using the pairwise structural alignments where sequence similarity falls between 10–20% (Fig 2, panels D-F), as this bin contains the highest number of pairwise alignments (see Fig 1). When correcting for solvent exposure, a clear, qualitatively similar, and highly significant difference is present between helix and strand residues in all four SCOP classes (Fig 2D–2F, S3 Fig). This indicates that, except for the most buried residues (RSA < 0.1), the same number of mutations result in less change in secondary structure in helices than strands, with coils being the least resilient against mutations.

In comparison with point mutations, the accumulation of indels shows a less clear pattern. The frequency of indels is significantly higher in helices than strands, although this is partly due to differences in solvent accessibility (S2 Fig, p < 2.2 x 10^−16^ ANCOVA). After correction for RSA, there is no qualitative difference in indel frequency in α/β domains (Supplementary Fig 2), although in all-α, all-β and α+β domains the frequency of indels is significantly higher in helices than strands (p < 2.2 x 10^−16^, ANCOVA).

### The higher robustness of helices is caused by their higher number of residue-residue interactions

**Fig 3:**
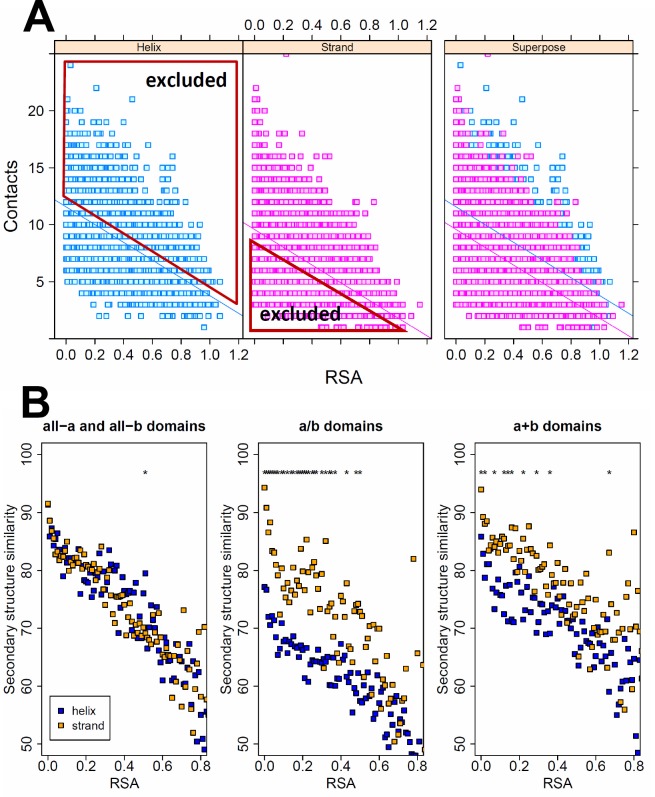
The effect of the number of residue contacts on secondary structure similarity. **A)** Residues in helices have significantly more non-covalent interactions than residues in strands (ANCOVA using all-α and all-β domains). Using the two regression lines between RSA and the number of inter-residue contacts of each residue, we excluded all helix residues with higher than average number of contacts, and strand residues with lower than average number of contacts, and subsequently determined secondary structure similarity with the remaining residues. **B)** When using the remaining residue sets in all four SCOP classes, the difference in robustness between alpha helices and beta strands disappears, or even reverses (stars indicate significant difference, tests of proportions, p < 0.05 after Holm-Bonferroni correction), indicating that the higher robustness of helices is caused by their higher contact density.

What mechanism may be responsible for the different robustness against mutations? We tested three hypotheses: first, as the number of non-covalent residue interactions (contacts) was suggested as the main cause determining the overall robustness of protein folds [10,11,14], we tested whether the number of inter-residue contacts in helices is higher than in strands. We identified all non-covalent interactions in the SCOP domains with the RINerator tool, and found that helix residues have a consistently higher number of contacts than strand or coil residues, when RSA is taken into account, and that strands have more contacts than coils (S4 Fig). Next, using ANCOVA, we identified those helix residues with less than the average RSA-normalized number of contacts (i.e. residues that fall below the regression line Fig 3A), and those strand residues with higher than the average RSA-normalized number of contacts (the residues that fall above the regression line, Fig 3A), and repeated the analysis using only these residues. The results indicate that the number of residue contacts is a key factor responsible for the higher robustness of helices: using these subsets of residues, the difference between helices and strands disappears, or even reverses (Fig 3B).

Second, we tested whether the observed higher evolutionary stability of helices is also present when amino acids are examined independently, i.e. whether it is a result of different amino acid composition of helices and strands [21], or it is a property of the secondary structure itself, and the same amino acids behave differently when they are part of helices or strands. Using the pairwise alignments where sequence similarity falls between 10–20%, we calculated the fraction of helix and strand forming amino acids with the same secondary structure in both sequences, for different levels of solvent accessibility (S5 Fig). The results indicate that the higher stability of helices is not simply due to different amino acid composition: all amino acids except cysteine and methionine are more likely to keep their secondary structure in a helix than strand when mutated (S5A–S5C Fig). Additionally, the same amino acids have significantly more residue-residue interactions when they are part of a helix than a strand (S6 Fig), further corroborating the hypothesis that the different amounts of residue interactions is responsible for the differences in robustness.

Third, we tested whether the linear distance between the contacting residues on the amino acid sequence contributes to the observed pattern. We assumed that contacts between more distant residues are more likely to influence the overall stability of a protein fold than short-range contacts, and thus disturbing them is more likely to be deleterious (i.e. having an effect on fitness of the organism). In helices, the majority of residue interactions are formed between residues located close to each other in sequence, typically within one helical turn. In contrast, strand residues are involved in more long-range contacts, so disrupting them might be more detrimental (both helices and strands form more contacts than H-bonds, and when one considers all contacts the difference between them is less pronounced than one would expect based on H-bonds alone). Surprisingly, our results indicate that the average distance of contacting residues is not a major contributor to the higher stability of helices relative to strands (S7A Fig). As expected, secondary structure similarity increases with the average contact distance of the residues (so residues forming longer-range contacts are less likely to change their secondary structure). However the trend is highly nonlinear and levels off above average contact distance of 20 residues. In addition, helix residues are more robust against mutations when residues with the same average contact distance are considered (at least below contact distance 20 residues). Finally, when only the subsets of residues with higher than average (strands), and lower than average (helices) number of contacts were used in the analysis (as shown in Fig 3), the pattern reverses (S7B Fig).

**Fig 4:**
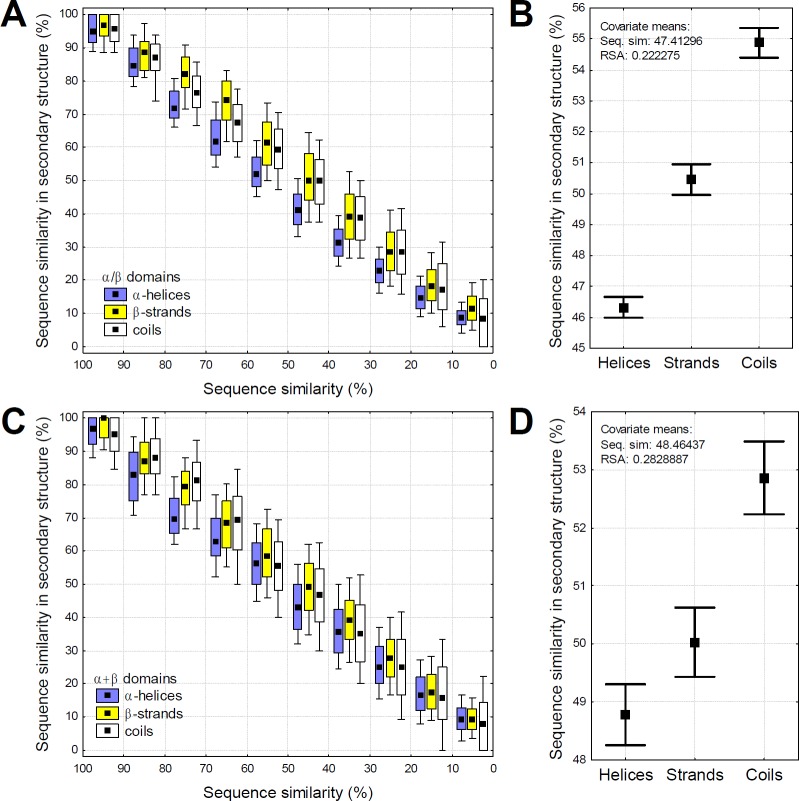
Within the same protein domains, helices diverge faster than strands, indicating higher robustness. **A)** The relationship between global sequence similarity and sequence similarity in secondary structures in α/β domains. The pairwise structural alignments were grouped into 10% bins (see Fig 2), boxes represent 25–75%, whiskers 10–90%. Note that the difference between helices and strands declines below 40% sequence similarity, because sequence similarity cannot be negative, and random sequences have an expected similarity of 5–6%. In the alignments with 10–90% sequence similarity, helices are significantly more diverged than strands and coils in each bin (p< 0.05, t-tests), also when the differences in their RSA is taken into account (p< 0.05, ANCOVA). **B)** An example of the independent effect of secondary structure on sequence divergence in α/β domains, using the pairwise structural alignments with 40–60% divergence, and ANCOVA with global sequence similarity and the average RSA of secondary structure as continuous predictors. Within the same domains, helices are significantly more diverged than strands (p < 2 x 10^−16^, whiskers represent 95% confidence intervals), which in turn are more diverged than coils (p < 2 x 10^−16^). **C-D)** The same as A-B, but for α+β domains.

Theoretically the lower rate of secondary structure change in helices can be the result of either stronger selection against change in helices or the higher robustness of helices. These two mechanisms however have contrasting predictions on the rate of sequence change. Residues and protein regions that can accept more mutations without a change are likely to accumulate mutations faster than regions that cannot, while regions under stronger purifying selection are likely to evolve more slowly. We tested whether the rate of sequence divergence within the same protein domains is different in helices, strands and coils. Our findings are in agreement with the hypothesis that more robust regions accumulate mutations faster: we observe a consistent trend that even within the same pairwise alignments, aligned helices are more diverged than strands, and coils are less diverged (Fig 4). This is also consistent with a report indicating that the evolutionary rate is higher in solvent exposed helices than in strands, and both evolve faster than coils [22].

The traditional view is that RSA is the most significant structural determinant of residue evolutionary rate in proteins and that the independent contribution of contact density is minor [20]. A few recent studies however have suggested that residue interactions (contact density) are more important [23,24], and the independent contribution of RSA is small. As contact density is highest in buried regions, which also evolve at the lowest rate, these studies are at odds with the findings reporting that designability/evolvability is positively correlated with the number of residue-residue interactions in a domain [10–12,14,25,26], and also with our findings here. It is not immediately clear to us what is the solution to this paradox, however our–admittedly simple—estimates of the independent effects of contact density and RSA on amino acid change indicate that, when both these factors are taken into account simultaneously, RSA is a much stronger predictor of amino acid change than contact density (S8 Fig), supporting the findings of Franzosa and Xia [20].

### Mutations that change secondary structure are more likely to be pathogenic

**Fig 5:**
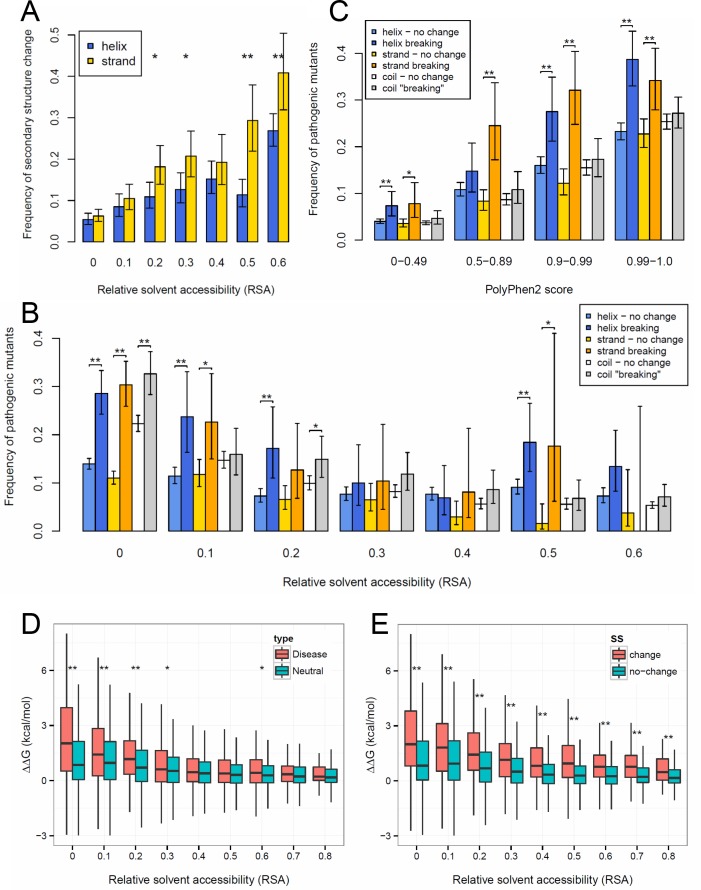
The effect of secondary structure on robustness and pathogenicity of point mutations. **A)** Point mutations in experimentally determined structures are significantly less likely to change secondary structure in helices than in strands. (On all panels “*” represents significance below 0.05 and “**” significance below 0.005, controlled for false discovery rate with the Benjamini-Hochberg method, error bars represent 95% confidence intervals.) **B)** The frequency of pathogenic mutants in conservative mutations that do not result in a change in secondary structure, and in secondary structure breaking mutations. Mutants were grouped according to the RSA of the wild type. Mutations that are predicted to break secondary structure are significantly more pathogenic than the ones that do not change secondary structure, particularly in the case of buried residues. **C)** Mutations with the same PolyPhen-2 (PP2) score are more likely to be pathogenic if they are predicted to change secondary structure, indicating that information on secondary structure can be used to improve pathogenicity prediction tools The numbers of mutations are 33492, 9953, 9082, 13369 for the PP2 score ranges 0–0.49, 0.5–0.89, 0.9–0.99, 0.99–1.0, respectively. **D)** Mutations that cause disease are significantly more destabilizing (have a larger effect on the free energy of folding) than neutral mutations in the RSA bins lower than 0.4. **E)** The higher pathogenicity of mutations that break secondary structure is probably caused by their stronger destabilizing effect on protein structure: the difference between secondary structure changing and non-changing mutations is highly significant in all RSA bins.

Given the above evolutionary analyses suggesting that helices are more robust to mutations than strands, we wondered whether this also could be related to observed patterns of human genetic and structural variation. First, to test whether the increased robustness of helices is still observed at the level of individual point mutations, we identified pairs of protein structures in the Protein Data Bank (PDB) that differ by individual amino acid substitutions (see Methods), and determined what fraction of point mutations change secondary structure. Similar to the pattern observed for evolutionarily diverged SCOP folds, we find that strands are much more likely to change secondary structure after a point mutation than helices, and the difference is particularly pronounced in the case of residues with high RSA (Fig 5A). We also observe that in the case of point mutations secondary structure changing mutations are located primarily at the ends of secondary structure units (see S9 Fig), and that with increasing solvent accessibility, a significantly higher fraction of mutations change secondary structure. However, this is likely to also be influenced by the fact that mutations that change secondary structure within the protein core are more likely to result in proteins that do not fold properly, and thus their structures are likely to be more difficult to crystallize.

Next, we identified 7199 human disease-associated missense mutations and 58 863 putative neutral mutations (see Methods) that could be mapped to experimentally determined structures present in the PDB. Since for the vast majority of these mutations structural data is available only for the wild type but not for the mutant, we used with PSSpred [27] and PSIpred [28] without the BLAST step to predict which mutations change secondary structure. We used only those predictions where PSSpred and PSIpred predicted the secondary structure of the wild-type correctly. Although the accuracy for predicting secondary structure changes is fairly low (see Methods), they do support our previous observation that helices tolerate mutations more than strands, as a significantly smaller fraction of mutations are predicted to break a helix than a strand or a coil (S10 Fig). Furthermore, the distribution of pathogenic mutations is significantly different in mutations that change secondary structure than in those that do not. Only a small fraction of mutations not predicted to break a helix or a strand is pathogenic (up to 15–20%, depending on relative solvent accessibility, Fig 5B). In contrast, mutations that result in secondary structure change are significantly more pathogenic (up to 2-fold in the case of mutations with RSA below 0.2, although the difference is declines with increasing RSA to nearly zero, Fig 5B).

Next, we investigated whether changes in secondary structure have utility for predicting pathogenicity. Currently the most commonly used tool to predict the pathogenicity of missense mutations is PolyPhen-2 (although some more recent tools, e.g. CONDEL [29], FATHMM [30] or SuSPect [31] seem to be more efficient), which utilizes both protein structural and evolutionary information [32]. We grouped mutations into four categories on the basis of their PolyPhen-2 scores: benign (0–0.49), possibly damaging (0.5–0.89), probably damaging (0.9–0.99) and highly likely damaging (0.99–1). Although, we did not find a clear difference in pathogenicity between helix and strand breaking mutations, we find that mutations predicted to change secondary structure are consistently more likely to be pathogenic, especially for the mutations that are predicted to be more damaging (i.e. have higher PolyPhen-2 score, Fig 5C). This is in line with the fact that some variant effect prediction tools utilize changes in secondary structure in prediction (SNAP [33]). Thus, despite the limited accuracy of the prediction of secondary structure changing mutations, it appears that information on predicted changes in secondary structure has considerable potential to improve pathogenicity predictions. For instance, a mutation predicted by PolyPhen-2 to be probably or highly likely damaging is almost twice as likely to be pathogenic if it is also predicted to change secondary structure. As PDB entries are not randomly selected proteins but are biased towards proteins with higher than average biological significance, having a PDB “hit” is itself a predictor of pathogenicity. To test whether in the absence of any structural information on the wild type secondary structure change can still be used to improve pathogenicity predictions, we repeated the above analysis for all human missense mutations, including the ones that cannot be mapped to PDB structure. As previously, we used PSSpred and PSIpred to predict mutations that change secondary structure, and we found a qualitatively similar pattern to the dataset that could be mapped to PDB: mutations that are predicted to change secondary structure are more likely to be pathogenic, particularly in the case of mutations with high PolyPhen-2 scores (S11 Fig).

Finally we examined whether the apparent pathogenicity of secondary structure breaking mutations is caused by their destabilizing effect on protein structure. For every mutation we calculated the difference between the free energy of folding of the wild type and the mutant (ΔΔG), using FoldX [34]. Similarly to a recent report [35], we found that disease causing mutations have a significantly more destabilizing effect on protein structure than neutral mutations, at least in the protein core (Fig 5D). The lack of significant difference in residues located close to the surface probably reflects the recent finding that a significant fraction of diseases with a genetic background is caused by modifications of protein-protein interactions, and not by erroneous folding [6]. Mutations that result in secondary structure change have a clear destabilizing effect on protein structure irrespectively of RSA (Fig 5E), which explains their higher pathogenicity. In contrast, secondary structure itself has only a small, although significant effect on ΔΔG (S12 Fig), mutations in coils are somewhat more destabilizing than in helices or strands, which is consistent with their lower robustness.

## Conclusions

The findings presented in this study strongly indicate that alpha helices can tolerate more point mutations than beta strands (and in consequence, are more designable). Besides providing basic insights on robustness and designability of proteins, our findings may also have important practical implications: we show that the effect of a mutation on secondary structure can be used to improve predictions of the phenotypic effects of missense mutations. Additionally, the results also suggest that engineering *de novo* all-alpha proteins should be easier than all-beta ones, as more sequences are likely to fold to the same topology, even ignoring the distribution of long-range contacts.

## Materials and Methods

### Analysis of SCOP domains

SCOP 1.75 domains were downloaded from http://scop.mrc-lmb.cam.ac.uk/scop. We used the Astral95 subset in the analysis, to remove redundancies. The secondary structure of the residues in each SCOP domain was determined with DSSP[36], relative solvent accessibility of residues was calculated as the quotient of their solvent accessible surface provided by DSSP, and the total surface area of the amino acid in Gly-X-Gly triples [37].

In the comparative analysis, we calculated all possible pairwise structural alignments between domains of the same SCOP families with TMalign [18]. (In consequence, families with one domain could not be used.) The choice of the structural aligner does not have a qualitative effect on the results, using the RCSB Protein Comparison Tool (with CE algorithm) instead of TMalign results in a similar pattern (see S13 Fig). We excluded all structural alignments with a template modeling (TM) score lower than 0.5 [38] (normalized with the longer sequence) as these are likely to represent different folds, and also domains shorter than 80aa, as they typically have very simple topologies [38]. In addition, we excluded the variable and constant domains of antibodies (b.1.1.1 and b.1.1.2) from all-β domains. Next, the raw pairwise alignments produced by TMalign were postprocessed: we removed the unaligned tails of the N- and C- termini, and the remaining unaligned fragments were refined with Rascal[39] (v1.34), which we modified to refine only those parts of the structural alignments that remained unaligned by TMalign, thus it did not modify the blocks that were correctly aligned using structures.

Secondary structure similarity in the alignments was calculated as the number of aligned (non-indel) positions with the same secondary structure divided by the total number of aligned positions being either helix, strand or coil in any of the two sequences (Fig 1). Indel frequency was calculated as the number of unaligned helix or strand residues, divided by the total number positions with helix or strand residues in the alignment. Gaps in the structures, and domains where residue numbering is not monotonously increasing were excluded from the analysis. All calculations were performed with in-house Perl scripts, available on request.

Non-covalent residue contacts were determined with RINerator [40,41]. In brief, RINerator first adds hydrogens to the protein structure with Reduce [42] (hydrogens are missing from most protein structures determined by X-ray crystallography), next using the van der Waals surfaces of all atoms, it determines the number and strength of contacts between residues. Residues are assigned as contacting if the van der Waals radiuses of any of their atoms are closer than 0.25Å, excluding covalent bonds.

### Analysis of PDB point mutants and human variation

We used the following procedure to identify structures that differ in point mutations in the PDB. First we filtered out redundant sequences, i.e. we kept only one sequence from entries with identical amino acid sequences. Next, using usearch [43] we performed an all vs. all blast search with a minimum 98% sequence identity requirement, to identify pairs of highly similar but not 100% identical sequences (excluding sequences shorter than 64 amino acids). The resulting sequence pairs were aligned with muscle [44], and the location of each mutation was determined in the pair. For each mutation, using DSSP we determined the RSA of the affected residues, and also whether it results in a change in secondary structure. Coil residues were defined simply as residues that are neither helix nor strand. Since the same mutations can be present in several, minimally different sequences, to remove redundancies, identical mutations in homologous sequences were counted only once, irrespectively of the combination of PDB entries they occur. In case of sequence pairs that differ in more than one residue, we used only the mutations that are separated by at least 10 residues in the sequences.

Data on human pathogenic and putative neutral missense mutations, including PolyPhen-2 pathogenicity predictions was downloaded from the Ensembl Variation database [45], release 83. Disease mutations were those annotated as pathogenic or likely pathogenic, whereas the putative neutral mutations were those with an assigned allele frequency, or annotated as benign. Although some of the low frequency variants are still likely to have a phenotypic effect or might be damaging [46], especially in a homozygous state, this gives us a large set mutations that should be highly enriched in those that are neutral or nearly neutral. Finally, given that many immune-related proteins are highly mutated, we excluded human proteins with immunoglobulin or HLA domains (PFAM families PF07686, PF08204, PF15910, PF07654, PF16196, PF05790, PF08205, PF07679, PF00047, PF00129). Only those mutants were included in the analysis where the wild type sequences have at least 90% sequence identity to a PDB structure and all side chain atoms for the wild type residue are observed in the structure. For each mutation, the RSA and secondary structure were determined with DSSP, using the structure. For those residues that mapped to multiple structures, we used the lowest RSA value.

To predict changes in secondary structure for the human variants, we used PSSpred [27] and PSIpred [28] without the PSI-BLAST step, and benchmarked them with the PDB point mutation dataset (see above). Previously, it has been shown that secondary structure prediction can reach up to 80–82% accuracy when applied to a complete protein sequences with a PSI-BLAST step and 65–69% without. Prediction accuracy is high for mutations that are predicted to be conservative and do not result in secondary structure change: 91% for helices, 92% for strands and 94% for coils. However we found that in the case of point mutations that are predicted to change secondary structure, current methods perform much worse. When used independently, prediction accuracy is only 30–24% for helices, 16–16% for strands and 15–14% for coils (PSSpred and PSIpred, respectively). By combining predictions from the two tools (i.e. using only the mutations for which PSSpred and PSIpred predictions are the same), the prediction accuracy is somewhat better, although still low: 38% for helices, 19% for strands and 18.7% for coils.

The effect of missense mutations on the free energy of folding (ΔΔG) was calculated with FoldX [34]. For every mutation we used the mapping PDB structure with the highest resolution, which was minimized with the FoldX RepairPDB utility prior to the ΔΔG calculation.

## Supporting Information

S1 Fig Histograms of relative solvent accessibilities (RSA) of helices and strands in the four main SCOP classes. Strands are significantly more buried in all classes, but the difference is particularly large in the case of α/β domains. This may be due to the fact that many α /β domains, such as TIM barrels, are typified by a central core of β strands surrounded by solvent accessible α helices.
(TIF)

S2 Fig Distribution of indels in the SCOP classes. The frequency of indels increases with decreasing sequence similarity (left panels), and is higher in helices than in strands in all-α and α+β domains, but not in α/β domains (right panels, using pairwise alignments with 10–20% sequence similarity).
(TIF)

S3 Fig Coils are less robust than helices or strands. The panels show data obtained from pairwise alignments with 10–20% sequence similarity, stars indicate significant difference between helices and strands (p < 0.05 after Holm-Bonferroni correction). While coils are clearly the less conserved, the biological interpretation of this pattern is not straightforward, because in the vast majority of cases helices and strands change independently from each other, while coils do not change independently from helices or strands: when a strand or helix residue changes to a coil, this is also counted as a change in coils. In consequence the amount of change in coils is close to the sum of the change in helices and strands.
(TIF)

S4 Fig Residues in alpha helices have more contacts than residues in beta strands, and strands have more contacts than coils in all four SCOP classes (ANCOVA, p < 2E-16). Boxes represent 25–75% intervals, whiskers 10–90%.
(TIF)

S5 Fig The higher robustness of helices is not the consequence of different amino acid composition, individual amino acids show the same trend. **A)** All-alpha vs. all-beta domains. Significantly different RSA bins are marked with stars. (Pairwise alignments with 10–20% sequence similarity, tests of proportions, significance level 0.05, corrected for multiple comparisons with the Holm-Bonferroni method.) **B)** α/β domains. **C)** α+β domains.
(PDF)

S6 Fig Individual amino acids have less non-covalent residue interactions in strands than in helices. **A)** Regressions between RSA and the number of contacts for glycine, in helices and strands (p < 2e-16 in all SCOP classes, ANCOVA). **B)** The difference between the intercepts of contacts-RSA regressions for helices and strands. The effect of secondary structure on the number of contacts is qualitatively the same in all amino acids: helices have a significantly higher number of contacts in all cases (p < 2e-16, ANCOVA).
(TIF)

S7 Fig The relationship between average contact distance on the protein sequence (i.e. the number of amino acids that separate two residues with a non-covalent residue interaction in the structure) and robustness to mutations, using the pairwise comparisons with sequence similarity between 10–20%. **A)** All residues **B)** Helix residues with low number of contacts and strand residues with high number of contacts (see Fig 3). Stars indicate significant difference between strands and helices (tests of proportions, p < 0.05, corrected for multiple comparisons with the Holm-Bonferroni method.)
(TIF)

S8 Fig RSA consistently predicts amino acid change better than contact density of residues (CD). The graph shows a scatterplot of RSA and CD importance, after bivariate binning. We selected all pairwise structural alignments of SCOP domains with sequence similarity between 45–55% (see Fig 1). For each pairwise alignment, RSA and CD were determined for every residue, and a logistic regression was made, to determine the relationship between the amino acid change, RSA and CD. Amino acid change was treated as a binary variable: when the aligned residues were identical the position was assigned 0, when not, 1. The “varImp” function of the “caret” R package was used to obtain the relative importance of the two predictors for each regression (on the scale of 0–100), which were then plotted, and binned in 2D with the “hexbin” R package, for clarity. While this approach is basic, and is not suitable to determine exact rates of amino acid change, it is sufficient to obtain a qualitative comparison of the importance of the two predictors. In general, both RSA and CD predict relatively poorly whether an amino acid will change or not, nevertheless RSA consistently outperforms CD.
(TIF)

S9 Fig Point mutations that break secondary structure are most frequently located close to the ends of secondary structure units. (TIF)

S10 Fig Missense mutations break helices less frequently than strands or coils. (* indicate a significant difference between helices and strands, corrected for multiple testing with the Holm-Bonferroni method. (p < 0.05, tests of proportions)
(TIF)

S11 Fig Secondary structure change can be used to improve PolyPhen-2 pathogenicity predictions, even if no structural information is available for the wild type, only predicted data. The pattern is qualitatively similar to Fig 5C, but the frequencies of pathogenic mutants are lower, because the PDB is biased towards proteins with pathogenic missense mutations: 50% of human pathogenic mutations that can be mapped to an experimentally determined structure, but only 10% of the neutral mutations. (Error bars represent 95% confidence intervals, “*” represents significance below 0.05 and “**” significance below 0.005 (tests of proportions), controlled for false discovery rate with the Benjamini-Hochberg method).
(TIF)

S12 Fig The effect of mutations on the free energy of folding (ddG) within different secondary structures. Stars indicate significant difference (*: p < 0.05; **: p < 0.005), after correction for false discovery rate (with the Benjamini-Hochberg method). Since the vast majority of mutations do not result in secondary structure change, secondary structure has only a small effect on ddG, nevertheless mutations in coils are consistently more destabilizing than mutations in strands or helices, except for the most buried residues with no solvent exposed area.
(TIF)

S13 Fig Using the RCSB Protein Comparison Tool (CE algorithm) for making the pairwise structural alignments results in an identical pattern as using TMalign—helices change less with sequence change. The panels show the same as panels D-F on Fig 2, but using the RCSB Protein Comparison Tool: alignments with 10–20% sequence similarity, with relative solvent accessibility (RSA) as a covariate.
(TIF)
