# Supplementary figures and images for: Alpha Helices Are More Robust to Mutations than Beta Strands
Source: PLoS Comput Biol. 2016 Dec 9;12(12):e1005242. doi: 10.1371/journal.pcbi.1005242 (PMC5147804; doi:10.1371/journal.pcbi.1005242)

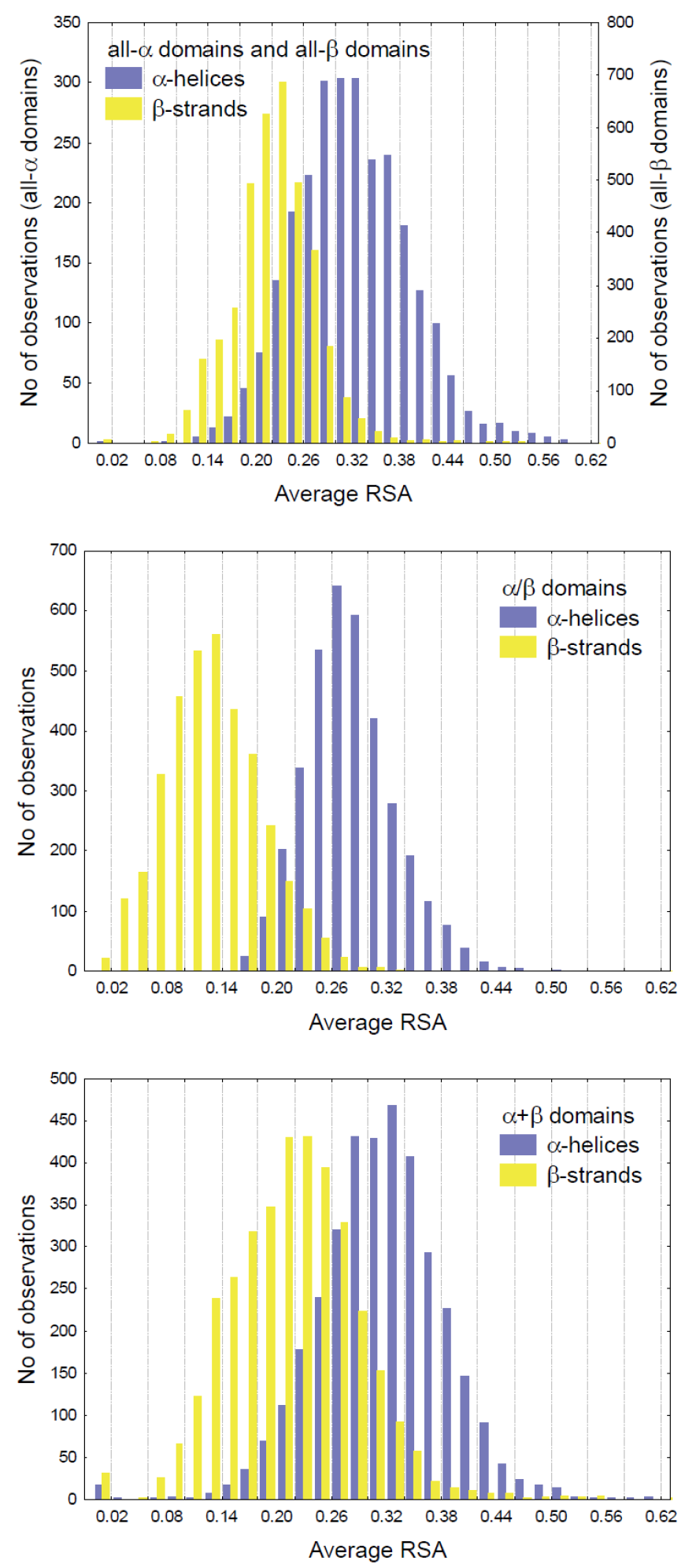

Supplement: S1 Fig — Strands are significantly more buried in all classes, but the difference is particularly large in the case of α/β domains. This may be due to the fact that many α /β domains, such as TIM barrels, are typified by a central core of β strands surrounded by solvent accessible α helices. (TIF) [file pcbi.1005242.s001.tif]

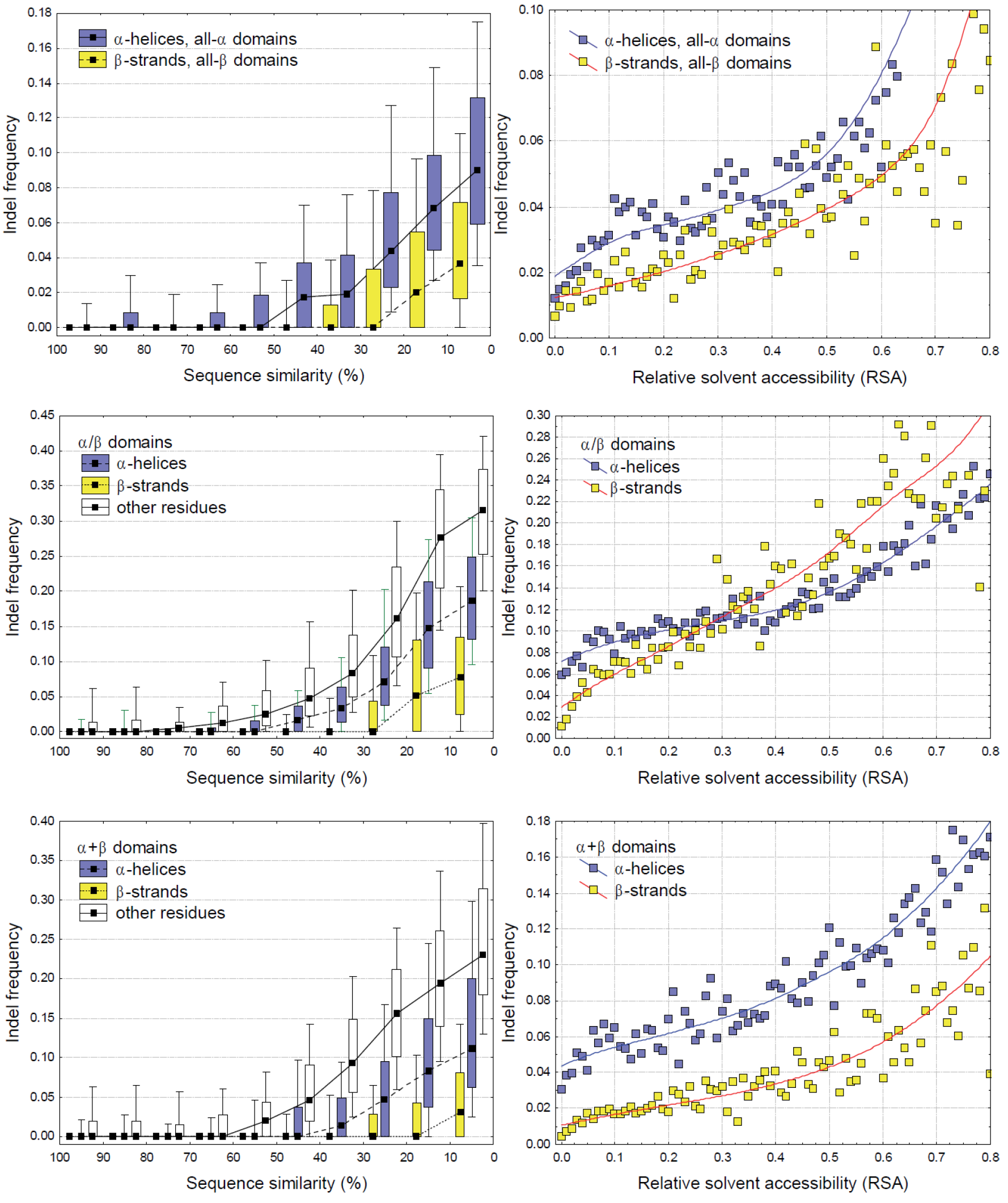

Supplement: S2 Fig — The frequency of indels increases with decreasing sequence similarity (left panels), and is higher in helices than in strands in all-α and α+β domains, but not in α/β domains (right panels, using pairwise alignments with 10–20% sequence similarity). (TIF) [file pcbi.1005242.s002.tif]

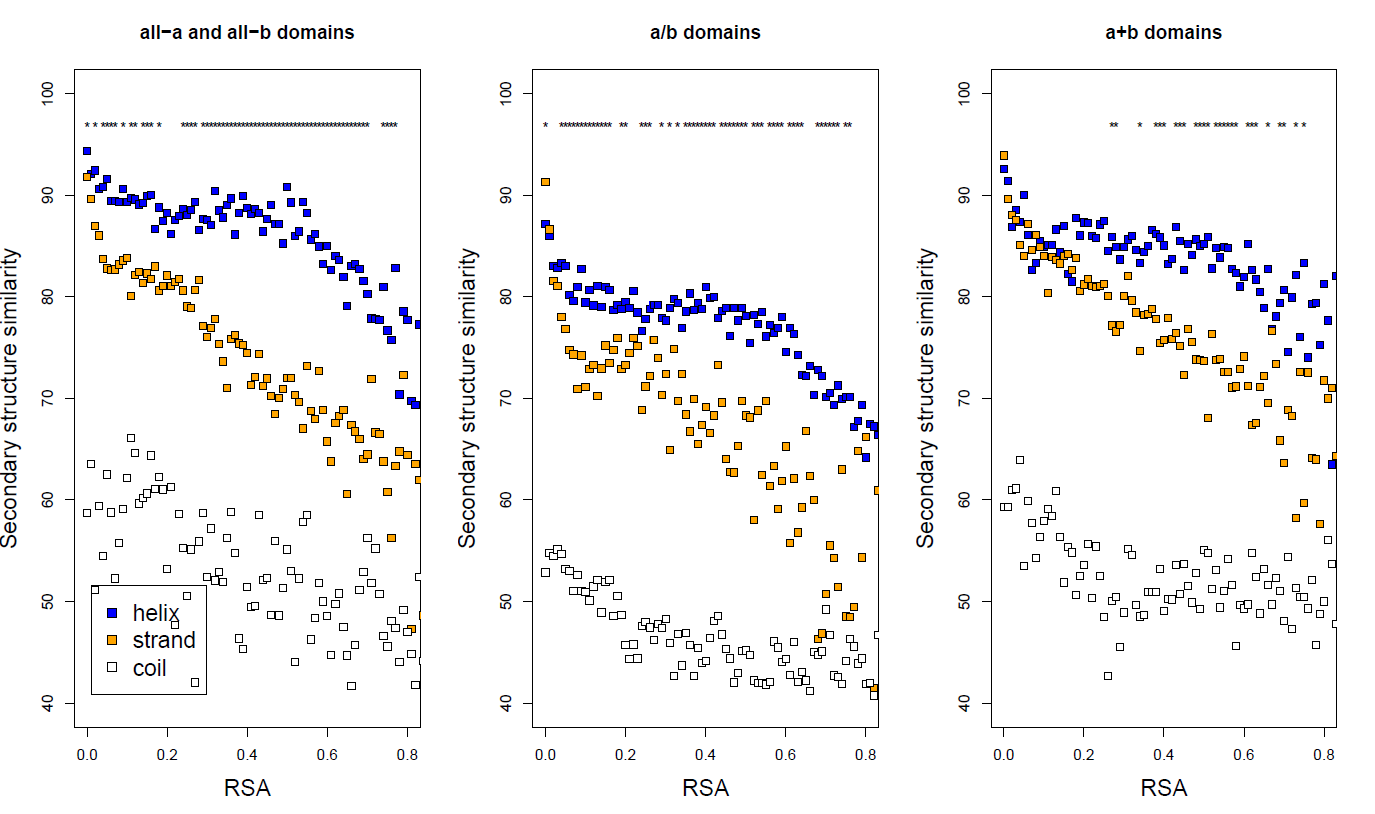

Supplement: S3 Fig — The panels show data obtained from pairwise alignments with 10–20% sequence similarity, stars indicate significant difference between helices and strands (p < 0.05 after Holm-Bonferroni correction). While coils are clearly the less conserved, the biological interpretation of this pattern is not straightforward, because in the vast majority of cases helices and strands change independently from each other, while coils do not change independently from helices or strands: when a strand or helix residue changes to a coil, this is also counted as a change in coils. In consequence the amount of change in coils is close to the sum of the change in helices and strands. (TIF) [file pcbi.1005242.s003.tif]

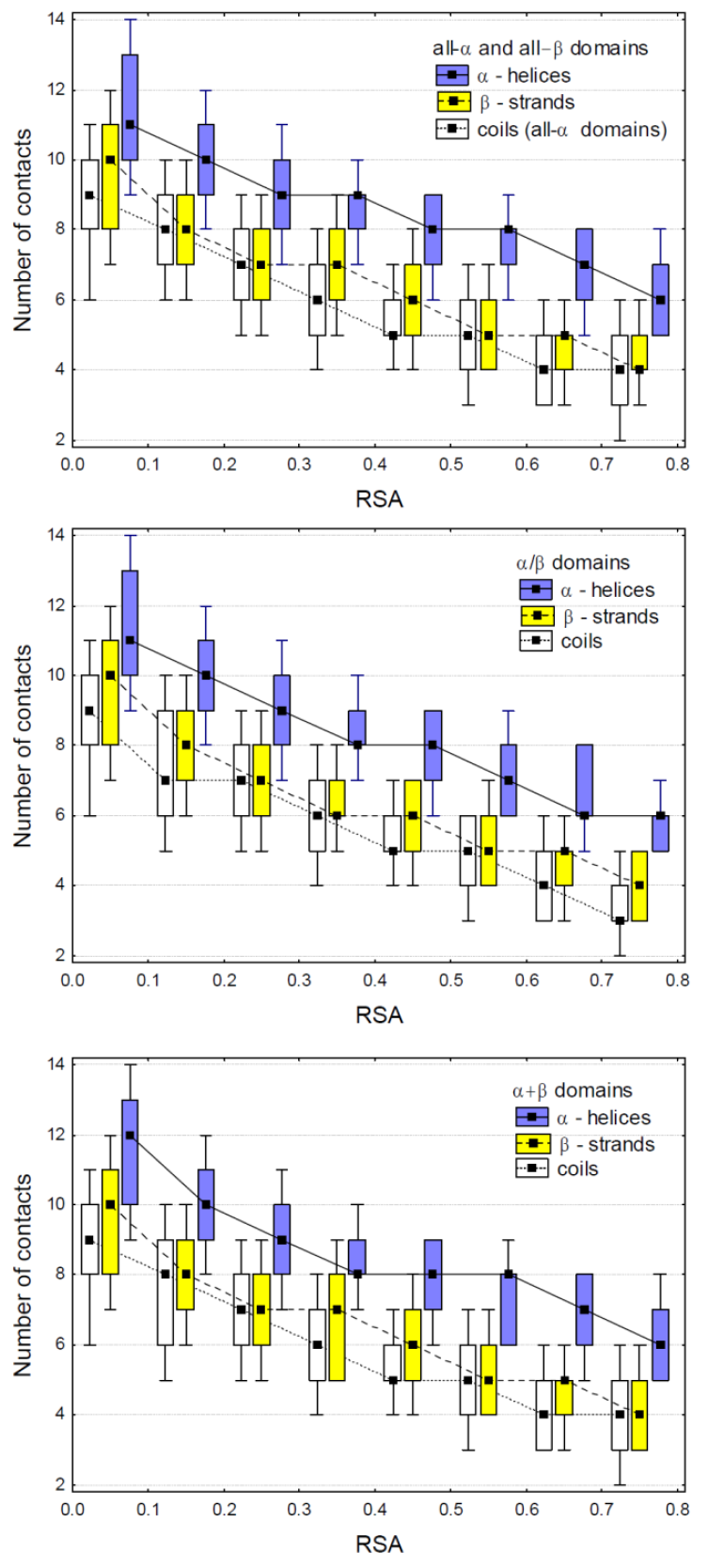

Supplement: S4 Fig — Boxes represent 25–75% intervals, whiskers 10–90%. (TIF) [file pcbi.1005242.s004.tif]

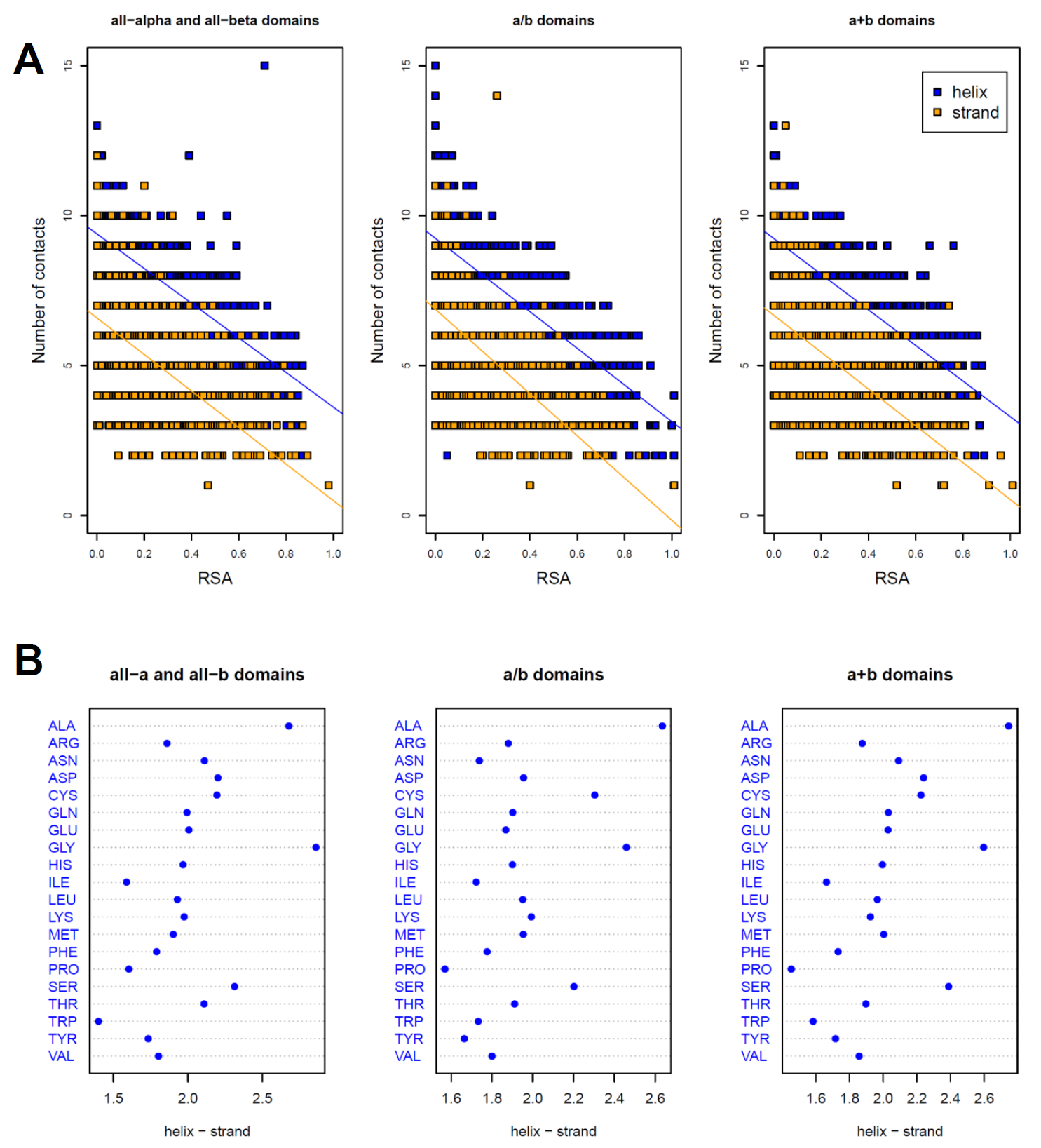

Supplement: S6 Fig — A) Regressions between RSA and the number of contacts for glycine, in helices and strands (p < 2e-16 in all SCOP classes, ANCOVA). B) The difference between the intercepts of contacts-RSA regressions for helices and strands. The effect of secondary structure on the number of contacts is qualitatively the same in all amino acids: helices have a significantly higher number of contacts in all cases (p < 2e-16, ANCOVA). (TIF) [file pcbi.1005242.s006.tif]

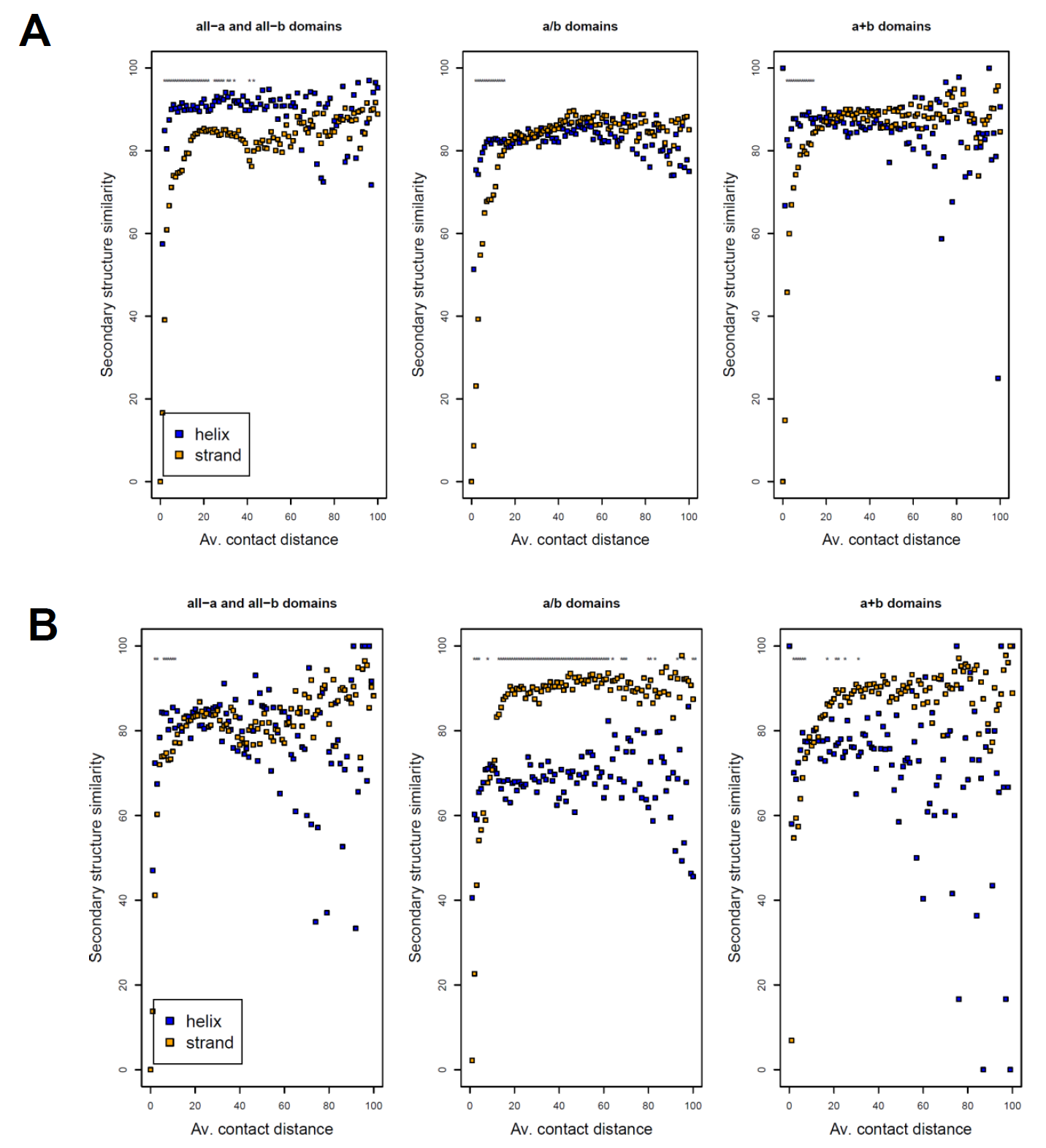

Supplement: S7 Fig — A) All residues B) Helix residues with low number of contacts and strand residues with high number of contacts (see Fig 3). Stars indicate significant difference between strands and helices (tests of proportions, p < 0.05, corrected for multiple comparisons with the Holm-Bonferroni method.) (TIF) [file pcbi.1005242.s007.tif]

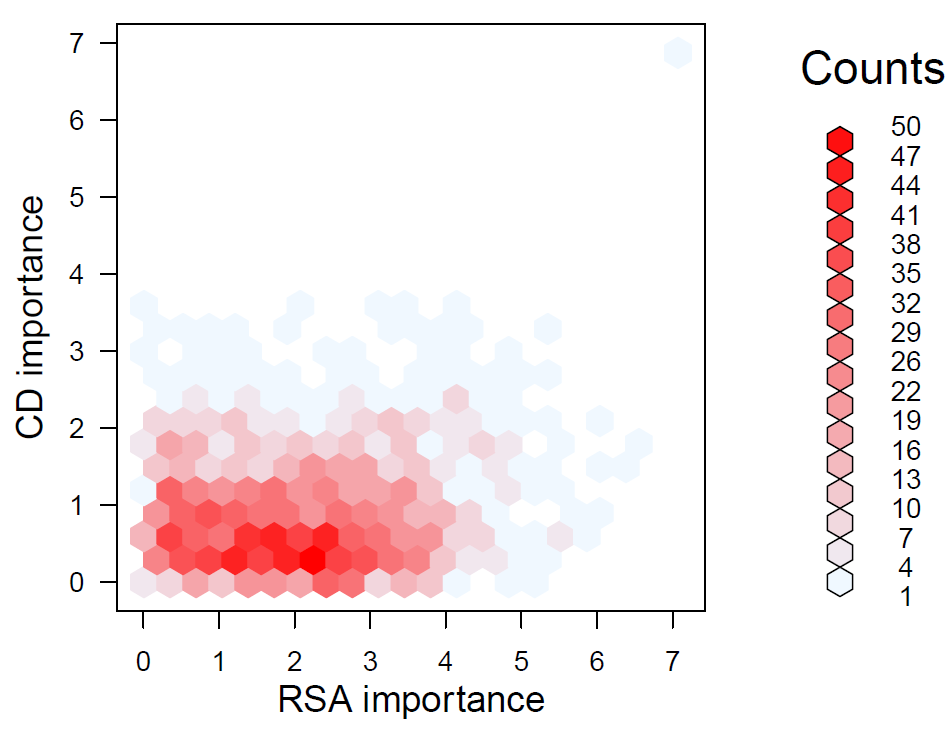

Supplement: S8 Fig — The graph shows a scatterplot of RSA and CD importance, after bivariate binning. We selected all pairwise structural alignments of SCOP domains with sequence similarity between 45–55% (see Fig 1). For each pairwise alignment, RSA and CD were determined for every residue, and a logistic regression was made, to determine the relationship between the amino acid change, RSA and CD. Amino acid change was treated as a binary variable: when the aligned residues were identical the position was assigned 0, when not, 1. The “varImp” function of the “caret” R package was used to obtain the relative importance of the two predictors for each regression (on the scale of 0–100), which were then plotted, and binned in 2D with the “hexbin” R package, for clarity. While this approach is basic, and is not suitable to determine exact rates of amino acid change, it is sufficient to obtain a qualitative comparison of the importance of the two predictors. In general, both RSA and CD predict relatively poorly whether an amino acid will change or not, nevertheless RSA consistently outperforms CD. (TIF) [file pcbi.1005242.s008.tif]

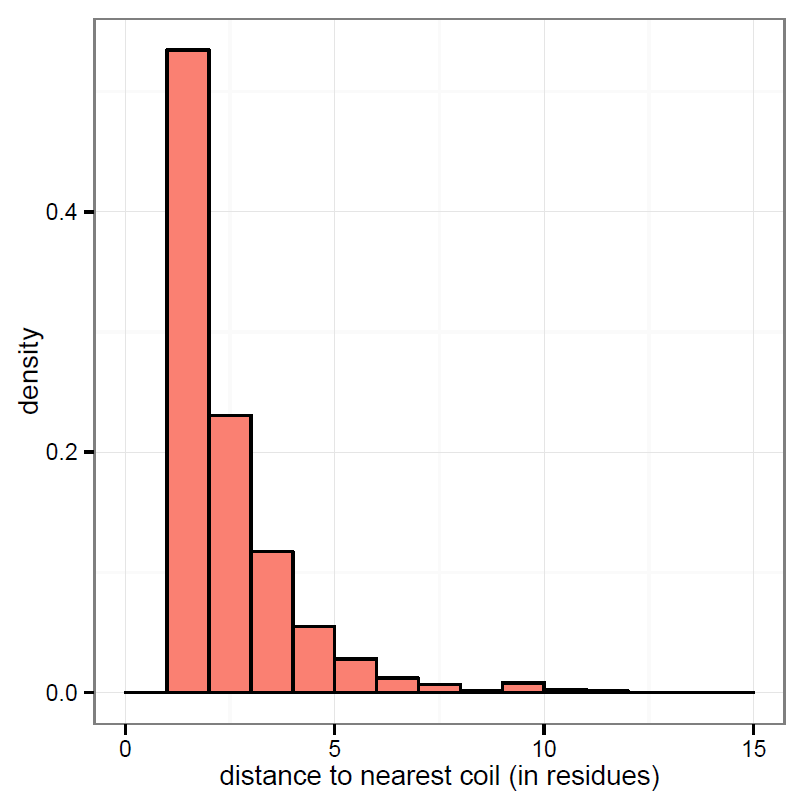

Supplement: S9 Fig — (TIF) [file pcbi.1005242.s009.tif]

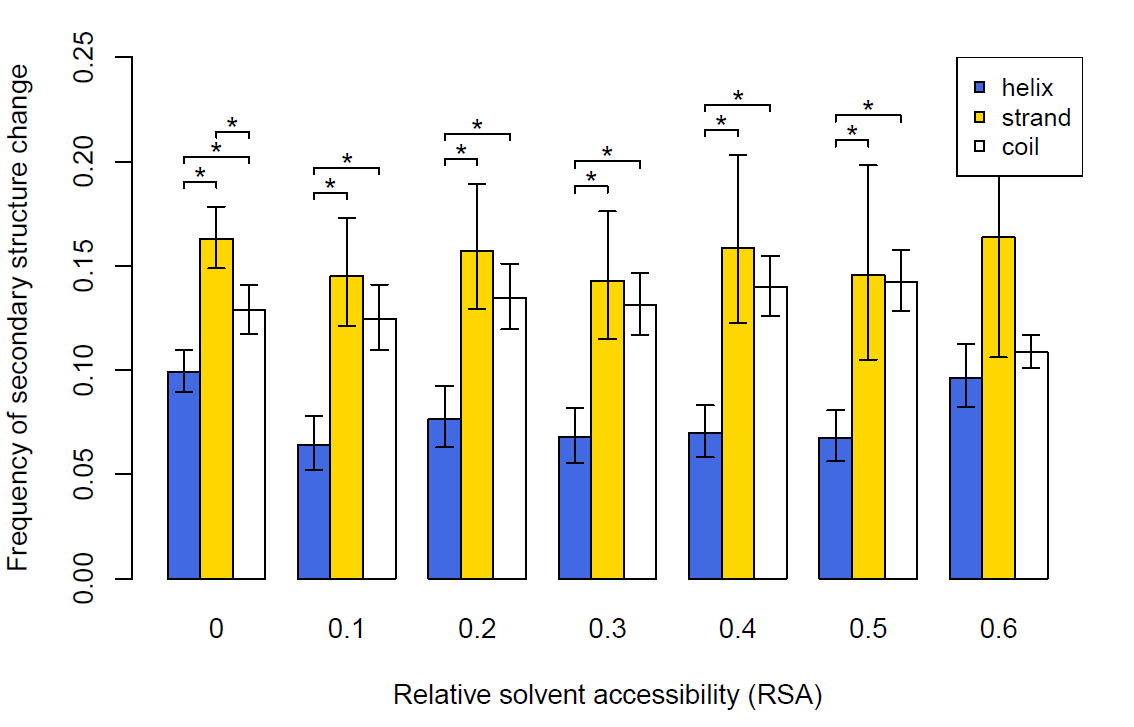

Supplement: S10 Fig — (* indicate a significant difference between helices and strands, corrected for multiple testing with the Holm-Bonferroni method. (p < 0.05, tests of proportions) (TIF) [file pcbi.1005242.s010.tif]

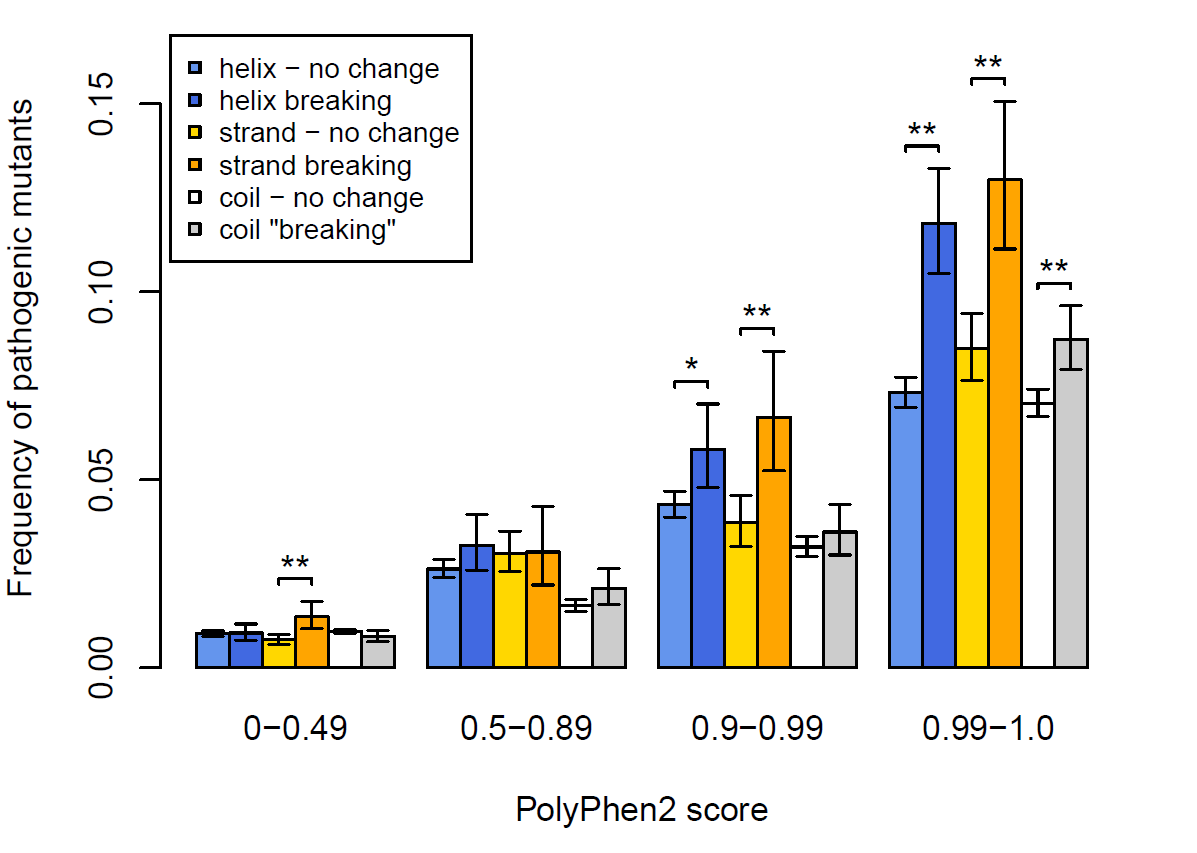

Supplement: S11 Fig — The pattern is qualitatively similar to Fig 5C, but the frequencies of pathogenic mutants are lower, because the PDB is biased towards proteins with pathogenic missense mutations: 50% of human pathogenic mutations that can be mapped to an experimentally determined structure, but only 10% of the neutral mutations. (Error bars represent 95% confidence intervals, “*” represents significance below 0.05 and “**” significance below 0.005 (tests of proportions), controlled for false discovery rate with the Benjamini-Hochberg method). (TIF) [file pcbi.1005242.s011.tif]

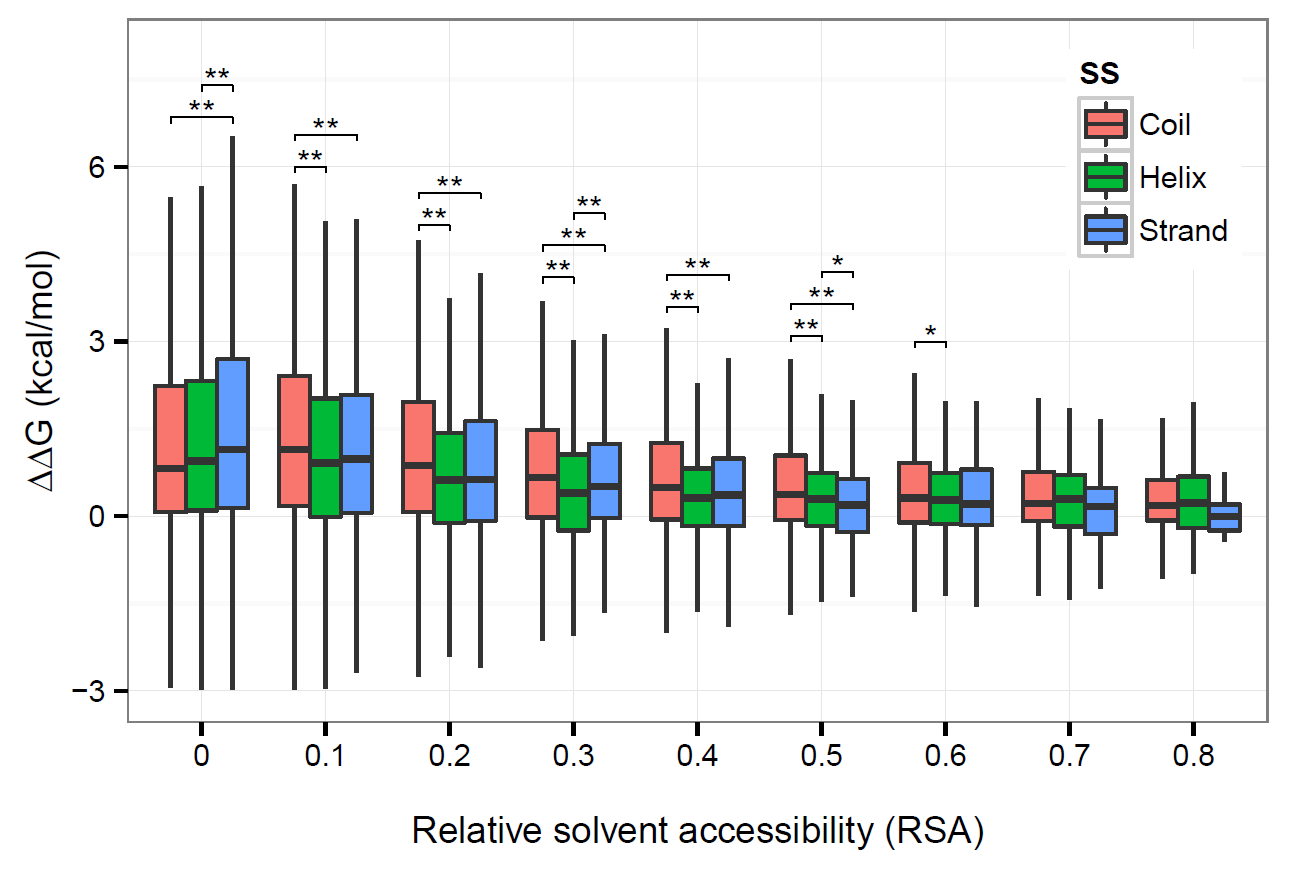

Supplement: S12 Fig — Stars indicate significant difference (*: p < 0.05; **: p < 0.005), after correction for false discovery rate (with the Benjamini-Hochberg method). Since the vast majority of mutations do not result in secondary structure change, secondary structure has only a small effect on ddG, nevertheless mutations in coils are consistently more destabilizing than mutations in strands or helices, except for the most buried residues with no solvent exposed area. (TIF) [file pcbi.1005242.s012.tif]

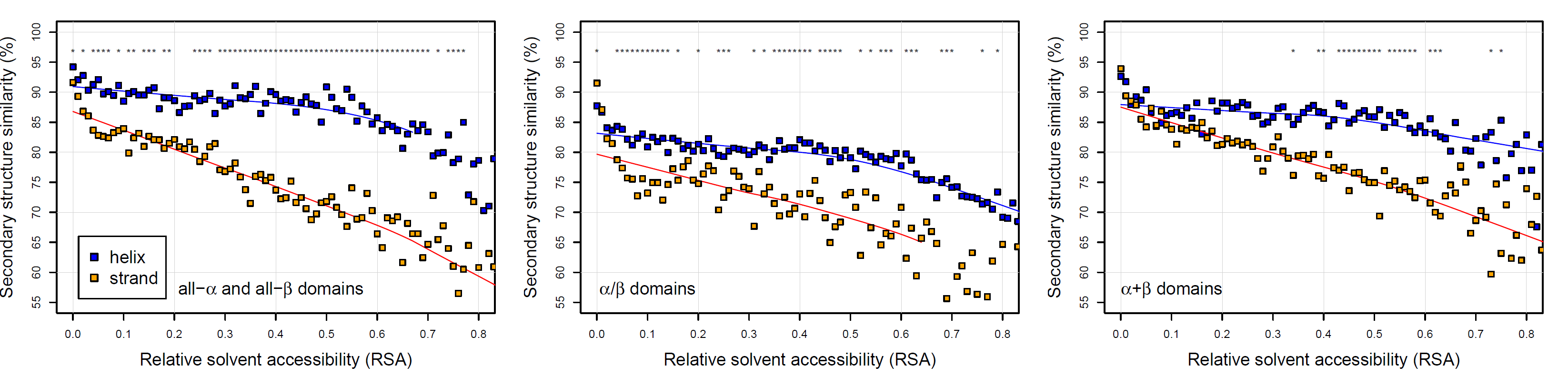

Supplement: S13 Fig — The panels show the same as panels D-F on Fig 2, but using the RCSB Protein Comparison Tool: alignments with 10–20% sequence similarity, with relative solvent accessibility (RSA) as a covariate. (TIF) [file pcbi.1005242.s013.tif]
