# Supplementary material for: Alpha Helices Are More Robust to Mutations than Beta Strands
Source: PLoS Comput Biol. 2016 Dec 9;12(12):e1005242. doi: 10.1371/journal.pcbi.1005242 (PMC5147804; doi:10.1371/journal.pcbi.1005242)
Supplement: S5 Fig — A) All-alpha vs. all-beta domains. Significantly different RSA bins are marked with stars. (Pairwise alignments with 10–20% sequence similarity, tests of proportions, significance level 0.05, corrected for multiple comparisons with the Holm-Bonferroni method.) B) α/β domains. C) α+β domains. (PDF) [file pcbi.1005242.s005.pdf]

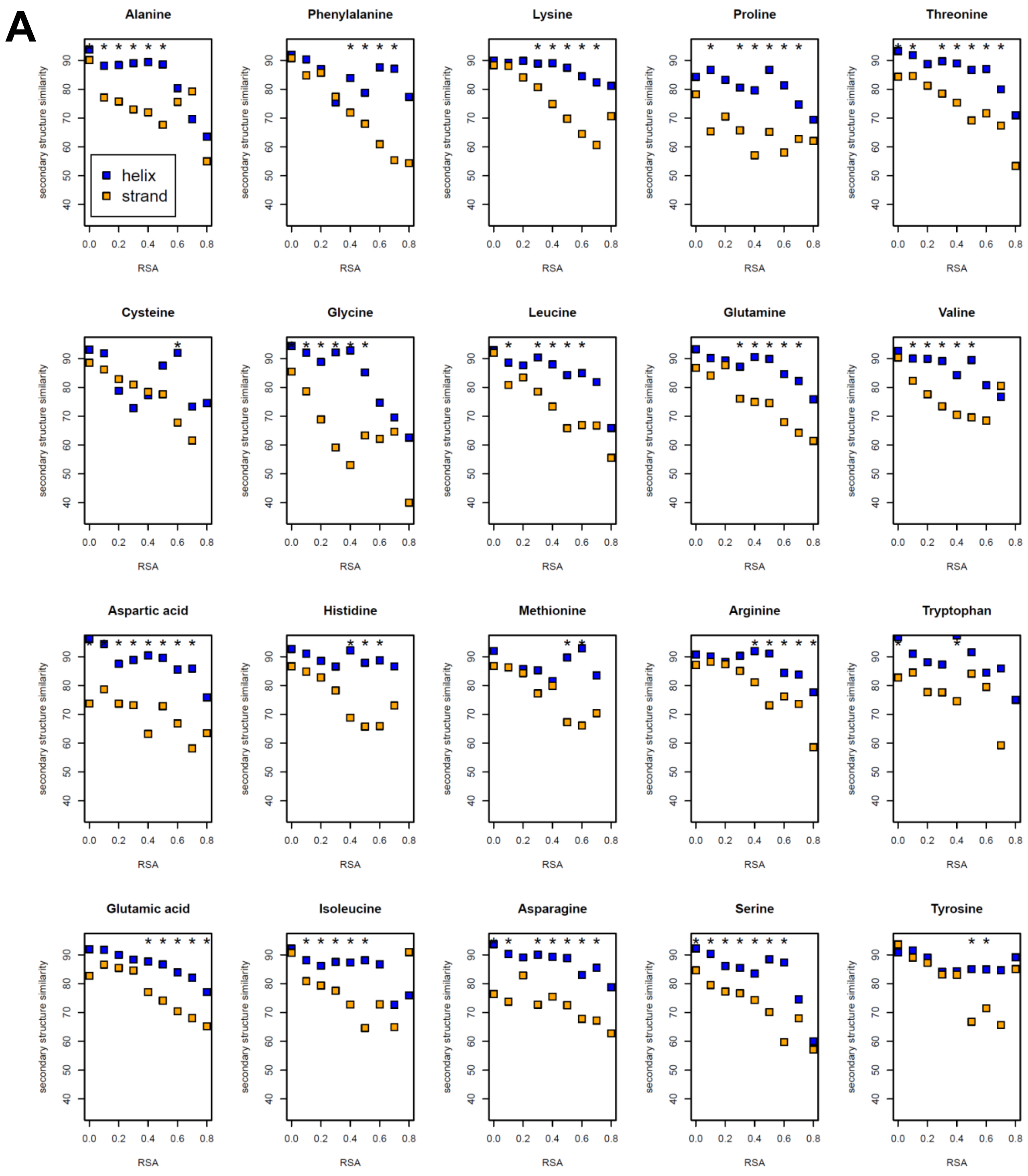

S5 Figure A) All-alpha vs. all-beta domains.

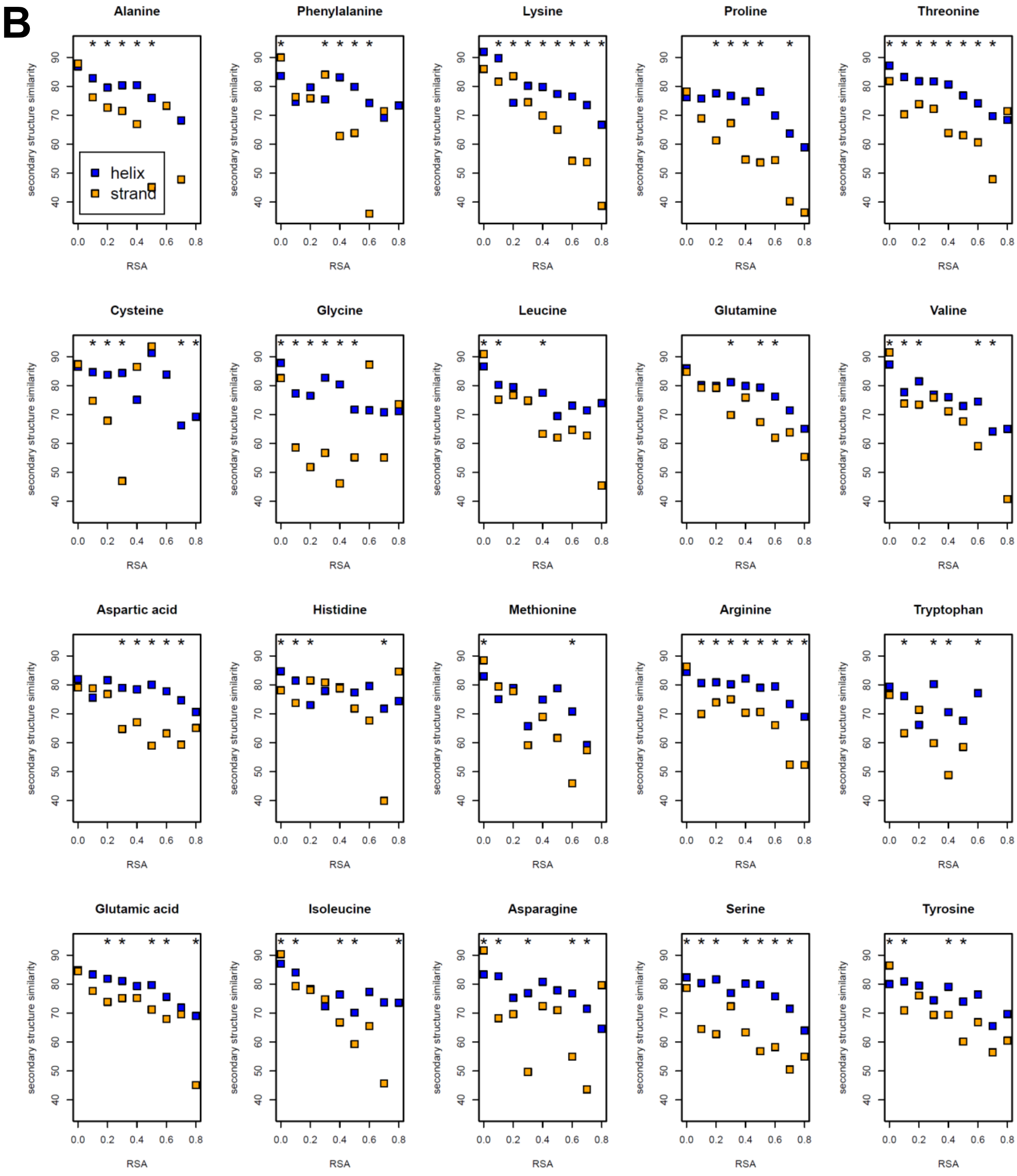

S5 Figure – continued. B) α/β domains.

C

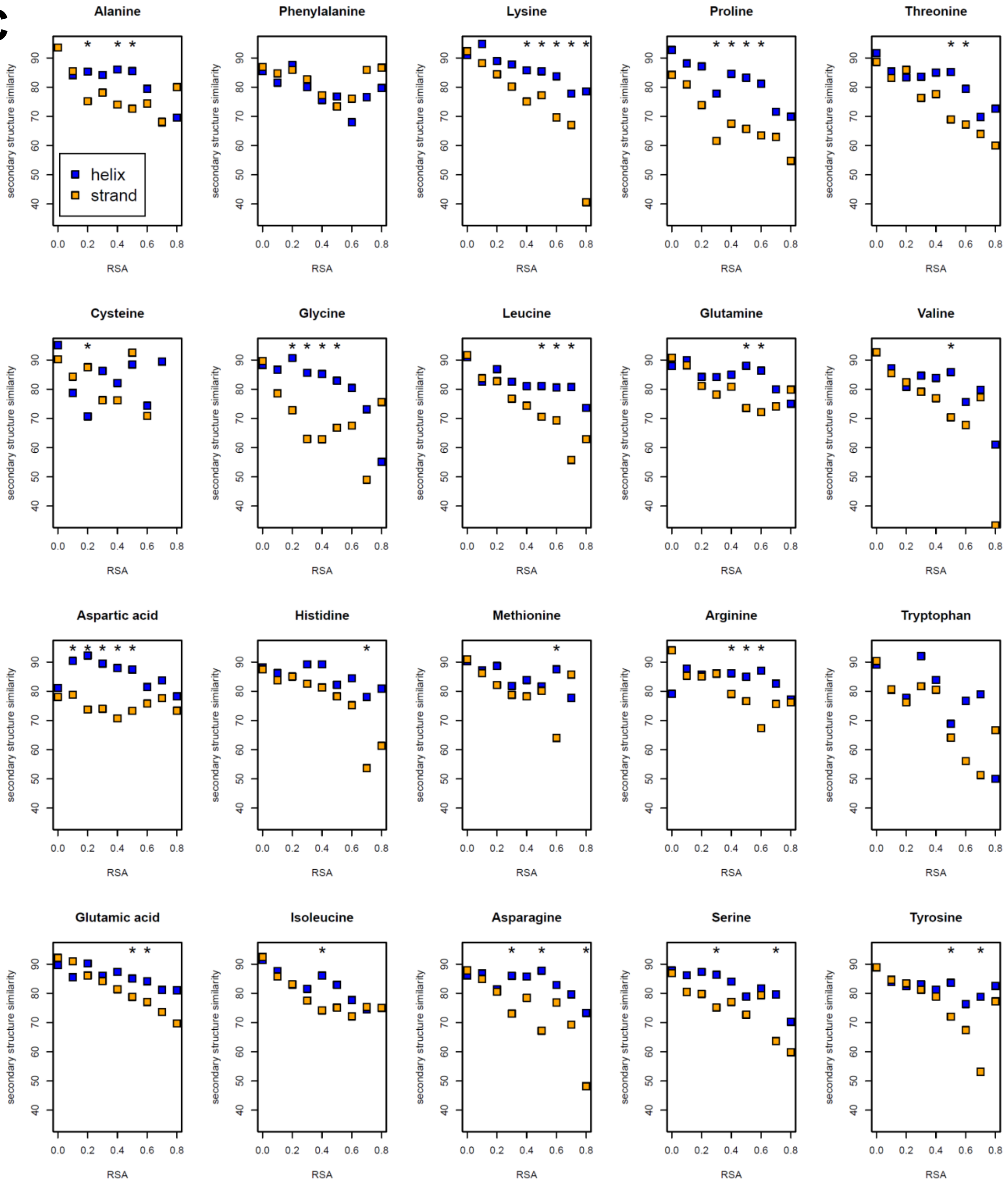

S5 Figure - continued. C) α+β domains.
